# CRISPR-Cas12a bends DNA to destabilize base pairs during target interrogation

**DOI:** 10.1093/nar/gkae1192

**Published:** 2024-12-19

**Authors:** Katarzyna M Soczek, Joshua C Cofsky, Owen T Tuck, Honglue Shi, Jennifer A Doudna

**Affiliations:** Department of Molecular and Cell Biology, University of California Berkeley, Berkeley, CA, USA; Innovative Genomics Institute; University of California, Berkeley, CA, USA; California Institute for Quantitative Biosciences (QB3), University of California, Berkeley, CA, USA; Department of Molecular and Cell Biology, University of California Berkeley, Berkeley, CA, USA; Innovative Genomics Institute; University of California, Berkeley, CA, USA; Innovative Genomics Institute; University of California, Berkeley, CA, USA; Department of Chemistry, University of California Berkeley, Berkeley, CA, USA; Department of Molecular and Cell Biology, University of California Berkeley, Berkeley, CA, USA; Howard Hughes Medical Institute, University of California Berkeley, Berkeley, CA, USA; Department of Molecular and Cell Biology, University of California Berkeley, Berkeley, CA, USA; Innovative Genomics Institute; University of California, Berkeley, CA, USA; California Institute for Quantitative Biosciences (QB3), University of California, Berkeley, CA, USA; Department of Chemistry, University of California Berkeley, Berkeley, CA, USA; Howard Hughes Medical Institute, University of California Berkeley, Berkeley, CA, USA; Gladstone-UCSF Institute of Genomic Immunology, San Francisco, CA, USA; Molecular Biophysics and Integrated Bioimaging Division, Lawrence Berkeley National Laboratory, Berkeley, CA, USA

## Abstract

RNA-guided endonucleases are involved in processes ranging from adaptive immunity to site-specific transposition and have revolutionized genome editing. CRISPR-Cas9, -Cas12 and related proteins use guide RNAs to recognize ∼20-nucleotide target sites within genomic DNA by mechanisms that are not yet fully understood. We used structural and biochemical methods to assess early steps in DNA recognition by Cas12a protein-guide RNA complexes. We show here that Cas12a initiates DNA target recognition by bending DNA to induce transient nucleotide flipping that exposes nucleobases for DNA-RNA hybridization. Cryo-EM structural analysis of a trapped Cas12a–RNA–DNA surveillance complex and fluorescence-based conformational probing show that Cas12a-induced DNA helix destabilization enables target discovery and engagement. This mechanism of initial DNA interrogation resembles that of CRISPR-Cas9 despite distinct evolutionary origins and different RNA-DNA hybridization directionality of these enzyme families. Our findings support a model in which RNA-mediated DNA interference begins with local helix distortion by transient CRISPR-Cas protein binding.

## Graphical Abstract

**Graphical Abstract:**
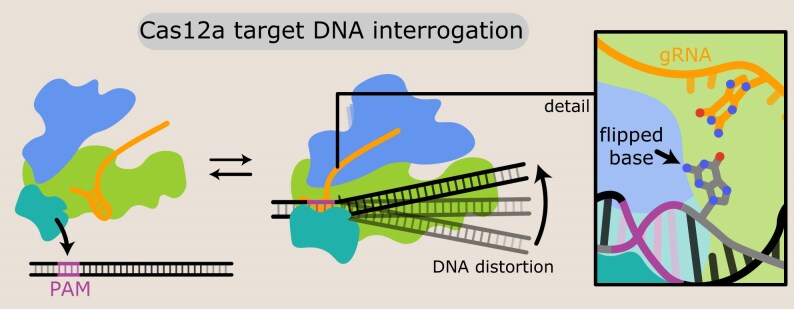

## Introduction

CRISPR-Cas12a uses guide RNAs to identify complementary ∼20-nucleotide sequences in genomic DNA to facilitate binding and double-strand cleavage (1). To engage DNA, Cas12a probes nucleotides immediately adjacent to 5′-TTTV-3′ protospacer adjacent motifs (PAMs) to test for guide RNA hybridization (1–3). When a sequence match is found, RNA–DNA binding forms an R-loop structure, enabling the RuvC nuclease domain to make staggered DNA cuts (1,2). In bacteria, double-stranded DNA breaks often induce DNA degradation, whereas in eukaryotic cells such breaks can trigger genome editing due to DNA repair (4,5).

**Figure 1.**
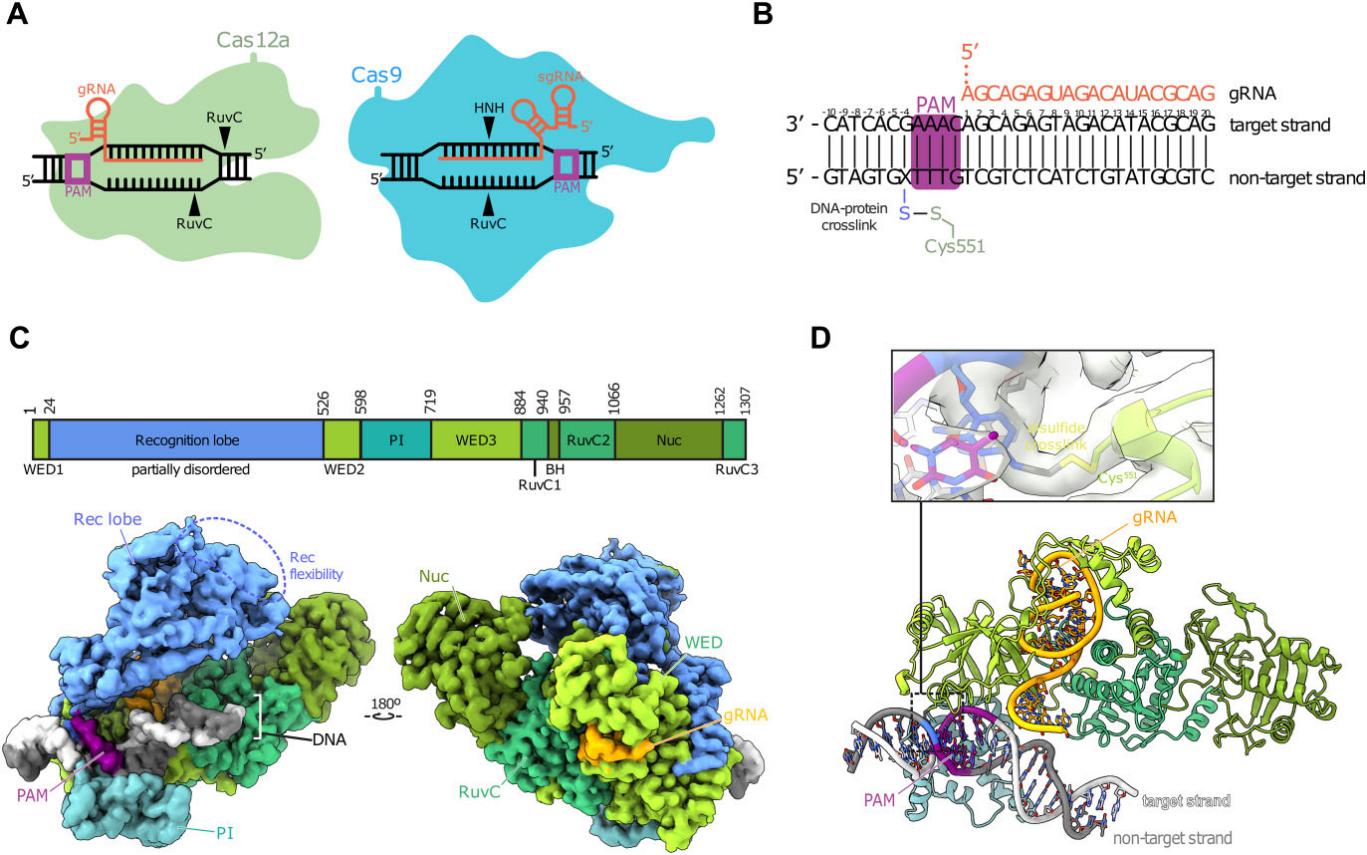
Disulfide crosslinking captures transient protein–DNA interactions. (**A**) Schematic representation of Cas12 (left) and Cas9 (right) R-loop complexes highlighting the differences between both enzymes. (**B**) DNA and guide RNA spacer sequences used in the study. The PAM sequence is highlighted in magenta; X shows the position of the cystamine modified base. (**C**) AsCas12a domain organization and cryoEM density of sharpened map of structure 2, map shown at 0.0125 level. (**D**) Model of structure 2 with detail centered on the disulfide bond crosslink between Cas12 and DNA. The Rec lobe is hidden for clarity.

The precise mechanism by which Cas12a locates target sequences amidst the vast excess of non-target sites in a typical genome remains unclear. Structural studies of both Cas9 and Cas12a revealed conformational changes that accompany R-loop formation but did not identify the initial steps of DNA engagement (6,7). Structural and biochemical studies of Cas9-guide RNA in complex with PAM-containing but otherwise non-target DNA provided evidence for DNA bending causing transient melting of the DNA sequence immediately adjacent to PAMs, enabling initial RNA-guided sequence interrogation (8). Because Cas9 and Cas12 evolved independently from different ancestral proteins (9,10), it has been unclear whether Cas12a functions by a similar mechanism (Figure 1A).

We used cryo-electron microscopy (cryo-EM) to determine structures of a complex between *Acidaminococcus* sp. Cas12a-guide RNA and a PAM-containing non target DNA molecule. Using site-specific cross-linking to trap the otherwise transient encounter between the Cas12a ribonucleoprotein (RNP) and the DNA, we observed three classes of DNA conformations with different degrees of bending relative to a standard B-form helix. In the most distorted class, a PAM-proximal target strand nucleotide is observed in two conformations, fully stacked as well as unstacked. The dynamic nature of this base was supported by fluorescence-based measurements of DNA conformation in solution. These data suggest that DNA bending induces base-flipping to enable Cas12a RNA-guided target recognition, a mechanism analogous to that of Cas9 despite independent evolutionary origins (8). Our findings help explain how Cas12a identifies target sites within genomes, a process that influences both the rate and outcomes of Cas12a-mediated genome editing.

## Materials and methods

### Protein expression and purification

Protein expression and purification were conducted as described in Cofsky *et al.* (11). Briefly, BL21 Star (DE3) *Escherichia coli* (Invitrogen) transformed with pJCC_099 (AsCas12a N551C) or pJCC_074 (AsCas12a WT) were grown in Terrific Broth (TB) and induced at mid-log stage growth with 0.5 mM IPTG overnight at 16°C. Cell pellets were resuspended in lysis buffer (50 mM HEPES (pH 7.5), 500 mM NaCl, 1 mM TCEP, 0.5 mM PMSF, 10 tablets/l cOmplete EDTA-free protease inhibitor cocktail (Roche), and 0.25 mg/ml chicken egg white lysozyme (Sigma-Aldrich) and sonicated. Lysate was centrifuged, and supernatant was loaded onto Ni-NTA Superflow resin (Qiagen). Resin was washed with wash buffer (50 mM HEPES (pH 7.5), 500 mM NaCl, 1 mM TCEP, 5% glycerol, 20 mM imidazole), and protein was eluted with elution buffer (50 mM HEPES (pH 7.5), 500 mM NaCl, 1 mM TCEP, 5% glycerol, 300 mM imidazole). TEV protease was added to the eluate, which was then dialyzed overnight against dialysis buffer (50 mM HEPES (pH 7.5), 250 mM NaCl, 1 mM TCEP, 5% glycerol). The sample was loaded onto MBPTrap HP and HiTrap Heparin HP columns (Cytiva) connected in series. After removing the MBPTrap column, the Heparin column was washed with ion exchange buffer A (50 mM HEPES (pH 7.5), 250 mM KCl, 1 mM TCEP, 5% glycerol) and eluted with a gradient of ion exchange buffer B (50 mM HEPES (pH 7.5), 1 M KCl, 1 mM TCEP, 5% glycerol). Peak fractions were concentrated and run in gel filtration buffer (20 mM HEPES (pH 7.5), 200 mM KCl, 1 mM DTT, 5% glycerol) over a HiLoad 16/600 Superdex 200 pg column (Cytiva). Peak elution fractions were pooled and concentrated to a final concentration between 40–80 μM.

### *In vitro* transcription of guide RNA

DNA templates for *in vitro* transcription were assembled by PCR from overlapping DNA oligonucleotides (IDT) (5′-GTCGAAATTAATACGACTCACTATAGG-3′, 5′-AATACGACTCACTATAGGTTTAATTTCTACTCTTGTAGAT-3′, 5′-CTGCGTATGTCTACTCTGCTATCTACAAGAGTAGAAAT-3′). The transcription reaction was assembled in transcription buffer (40 mM Tris–Cl (pH 7.9 at 25°C), 25 mM MgCl_2_, 10 mM dithiothreitol, 0.01% (v/v) Triton X-100, 2 mM spermidine, 5 mM each NTP, 100 μg/ml T7 RNA polymerase) and allowed to proceed at 37°C for 2.5 h. DNase I was added, and the sample was incubated for an additional 30 min at 37°C. RNA was then purified by denaturing PAGE (10% acrylamide:bis-acrylamide 29:1, 7 M urea, 0.5× TBE), ethanol-precipitated and resuspended in RNA storage buffer (0.1 mM EDTA, 2 mM sodium citrate, pH 6.4).

### DNA oligonucleotide preparation

The cystamine-functionalized DNA oligonucleotide (5′-GTAGTGXTTTGTCGTCTCATCTGTATGCGTC, where X denotes the N4-cystamine-2′-deoxycytidine) was synthesized by TriLink Biotechnologies. All other oligonucleotides were obtained from Integrated DNA Technologies. DNA oligonucleotides were purified in-house on urea-PAGE gels. DNA duplexes were annealed in water by heating to 95°C for 2 min and then cooling to 25°C over a 40 min period. For verification of cleavage activity upon crosslinking, extended cystamine-functionalized dsDNA was prepared as follows. A labeled oligonucleotide with a 10 nt extension (5′-6FAM-ATCTGACCATGACGCATACAGATGAGACGACAAAGCACTAC, where 6FAM denotes 6-carboxyfluorescein) was annealed with the cystamine functionalized oligonucleotide. The gel-purified product was extended by reaction with the Klenow Fragment (3′ to 5′ exo-, New England Biolabs) according to the manufacturer's protocol and was then isolated by phenol-chloroform extraction.

For oligonucleotide radiolabeling, T4 polynucleotide kinase (New England Biolabs) at 0.2 U/μl (manufacturer's units) was mixed with 1× T4 PNK buffer (New England Biolabs), 400 nM DNA oligonucleotide and 200 nM [γ-^32^P]-ATP (PerkinElmer) and incubated for 30 min at 37°C, then 20 min at 65°C. Radiolabeled oligo was then buffer exchanged into water using a Microspin G-25 spin column (GE Healthcare).

### Complex preparation

For crosslinking optimization, 6 μM functionalized DNA duplex, 5 μM RNA, and 4 μM AsCas12a were combined in cross-linking buffer (50 mM Tris pH 7.4, 150 mM NaCl, 5 mM MgCl_2_, 5% glycerol, 100 μM DTT). The reaction was incubated at 25°C for 12 h and then quenched by addition of S-methyl methanethiolsulfonate to a final concentration of 20 mM. Reactions were then denatured by addition of non-reducing SDS loading solution and heating for 5 min at 90°C.

For cryo-EM sample preparation, reactions were prepared as above but without quenching. After the 12-h incubation, the sample was injected onto a Superdex 200 Increase 10/300 GL column equilibrated with complex buffer (20 mM Tris pH 7.5, 200 mM KCl, 5 mM MgCl_2_, 0.25% glycerol and 100 μM DTT). Peak fractions were collected, concentrated and frozen at –80°C for storage.

For 2-aminopurine assays, reactions comprising 1.3 μM Cas12a, 1.7 μM RNA and 1 μM DNA in the crosslinking buffer were incubated overnight at 25°C to facilitate crosslinking.

### DNA cleavage assay

Reactions were conducted in DNA cleavage buffer (20 mM Tris pH 7.9, 150 mM KCl, 5 mM MgCl_2_, 5 mM TCEP). Reactions were initiated by adding DNA (non-target strand 5′-GTCATAATGATTTTATCTTCTGGATTGTTGTAAGCAGCATTTGAGCAAAAATCTGTTGC, target strand 5′-GCAACAGATTTTTGCTCAAATGCTGCTTACAACAATCCAGAAGATAAAATCATTATGAC) to AsCas12a:guide RNA complex, yielding final concentrations: 100 nM AsCas12a, 120 nM guide RNA, 1 nM radiolabeled DNA duplex. Reactions were quenched by addition of one volume of 2× quench buffer (94% formamide, 30 mM EDTA, 0.025% w/v bromophenol blue) and analyzed by denaturing PAGE (15% acrylamide bis-acrylamide 29:1, 7 M urea, 0.5× TBE) and phosphorimaging.

To confirm cleavage activity after crosslinking, a procedure reported in Cofsky *et al.* (8) was followed. In brief, 75 μl reactions were constructed in magnesium-free reaction buffer (50 mM Tris–Cl, pH 7.4, 150 mM NaCl, 1 mM EDTA, 5% glycerol, 100 μM DTT) with 60 μM cystamine dihydrochloride (pH 7), 4 μM AsCas12a WT or N551C, 5 μM complementary crRNA and 20 nM extended fluorophore-labeled cystamine DNA. After equilibration at 25°C for 2 h, S-MMTS was added to 20 mM for nonreducing conditions (bringing the total reaction volume to 80 μl), and DTT was added to 5 mM for reducing conditions (bringing the total reaction volume to 80 μl). Samples were incubated at 25°C for 5 min, then at 16°C for 15 min. At this point, aliquots were quenched in nonreducing SDS-PAGE solution and subject to SDS-PAGE in stain-free gels (mini-protean, Bio-Rad) to determine the extent of crosslinking. After zero time-point aliquots were quenched in a 2× quench buffer, reactions were initiated by addition of MgCl_2_ to a final concentration of 5 mM. Aliquots were quenched at the indicated time points for urea-PAGE analysis as described above but with the addition of 5% beta-mercaptoethanol before loading to reduce disulfide crosslinks. Fluorescein-labeled oligonucleotide cleavage was imaged in an Amersham Typhoon (Cytiva) in the Cy2 channel.

### EM grid preparation and data collection

Sample aliquots were thawed and diluted into complex buffer to a concentration of 3 μM. Grids (1.2-μm/1.3-μm 400 mesh C-flat grids, Electron Microscopy Sciences #CF413-50) were glow discharged (PELCO easiGlow) for 15 s at 25 mA. 3.6 μl of sample was applied to a grid in an FEI Vitrobot Mark IV operated at 8°C and 100% humidity. Excess sample was blotted for 3 s with blot force 6 before plunge freezing. Micrographs were collected on a Talos Arctica transmission electron microscope operated at 200 kV and ×36 000 magnification (1.115 Å per pixel), with −0.8 to −2 μm defocus, using the super-resolution camera setting (0.5575 Å per pixel) on a Gatan K3 Direct Electron Detector. 1684 movies were collected using beam shift in SerialEM v.3.8.7 software.

### Cryo-EM data processing

Initial data processing was conducted using cryoSPARC v.4 (Structura Biotechnology Inc.) (12). After motion correction (Patch Motion) and CTF estimation (Patch CTF), 1364 micrographs were curated for further processing. Initial particle picking was done using blob picker on a small subset of micrographs to generate templates that were used (Template Picker) to pick 1 929 565 particles from all micrographs. After two rounds of 2D classification, 459 736 particles remained. Particles were re-extracted with re-centering and used for *ab initio* reconstruction into five classes. Only one class corresponded to the expected complex (137 851 particles). Particles from this class were again re-extracted with re-centering, and subjected to non-uniform refinement, which provided a map with global resolution of 3 Å. In the sharpened map the DNA density was absent. Particles from the non-uniform refinement were transferred to RELION v. 5.0-beta (13) for DNA-focused refinement. After 3D refinement in RELION, signal from the protein was subtracted with a low pass filtered mask (10 Å) for the DNA fragment with a map extension of 4 pixels and a soft-edge over 6 pixels. The particles were re-centered on the DNA mask with a new box size of 120 pixels. Five DNA volume classes were reconstructed in 3D classifications without alignment with a T value of 15, a particle mask diameter of 110 Å and a volume mask where the input was low pass filtered to 5 Å, with a map extension of 3 pixels and a soft-edge over 5 pixels. Three classes, with 20, 031, 27, 147 and 27, 308 particles respectively, were chosen for final reconstruction. Particle subtraction was reversed to yield reconstructions of the full particles. Reconstructions were refined with masking of the volume that was low pass filtered to 10 Å, with a map extension of 4 pixels and a soft-edge over 6 pixels, solvent-flattened FSC and Blush (14) regularization and a particle mask diameter of 200 Å. Refined maps were sharpened (*B*-factor –30) and a mask was obtained with parameters as in the refinement, reaching nominal resolutions of 3.6 Å for structure 1 (S1), 3.4 Å for structure 2 (S2) and 3.5 Å for structure 3 (S3). These maps were used for initial model building and refinement. We then used DeepEMhancer (15) on refined maps with the default tightTarget model for maps S1 and S2, and highRes model and manual noise statistics (mean and standard deviation) obtained in Chimera X using the *volume statistics* tool for the S3 map. This improved the interpretability of our maps, which were used for final model building and refinement.

### Model building

The AsCas12a crystal structure (PDB 5B43) was used as the starting model. The initial model was built in Coot (v. 0.9.8.93 EL) (16) where each protein domain was fit separately into the density with manual adjustments. Both RELION sharpened and unsharpened maps were used in this process. The protein model was iteratively refined in Phenix (v. 1.19.2-4158) (17–19) using phenix.real_space_refine (minimization_global, local_grid_search, adp) with secondary structural restraints and manual geometry adjustments in Coot. Regions where neither the sharpened nor unsharpened map provided sufficient density remained unmodeled. Overall, the REC domain had the weakest density in the structures. The RNA sequence was adjusted to reflect our construct sequence. DNA was created as a linear B-form helix in Coot. PHENIX (19) simulated_annealing with base pair and base stacking restraints was used to fit the DNA into the density. In structure 3, near the PAM, we observe a weak density outside of the double stranded region that is connected to the DNA helix. In this region we built the nearby base in two conformations, one that is fully stacked and second that is unstacked and rotated away from the helix. In the unstacked conformation, we placed the base in the density, but the rest of the nucleotide has a poor fit to the map, suggesting that this density may represent a very transient state. For the flipped base, base pairing restraints were removed as well as stacking constraints between this base and upstream and downstream bases in both DNA chains. UCSF Chimera X ISOLDE (20) was used to improve the model of the PI and REC domains of Cas12a. Planarity was enforced on nucleotides within the DNA duplex in the protospacer region (5 base pairs; chain C bases 14–16 or 14–18 depending on the length of DNA) for all structures. The thioalkane linker was added as an ethanethiol ligand from PHENIX’s eLBOW module (21). Sulfur-sulfur and carbon-nitrogen bonds were restrained as described (8). Final protein models were built to the DeepEMhanced maps, where improved connectivity in the REC domain allowed us to dock larger fragments from the crystal structure. REC 1 (residues 24 – 323) and REC 2 (324- 523) were aligned with the starting model using Coot SSM superpose, and then their fit to the density was improved with jiggle fit. Region 265–323 was unaccounted for in our density and was removed. At the points where the starting model and rigid body fit REC domain were the closest, the models were merged (REC 1 residues 47–264, REC 2 residues 324–520 for structure 1 and structure 2, and REC 1 residues 53–264 and REC 2 residues 324–510 for structure 3). Geometry issues that appeared near the merge sites were resolved in Coot with ‘*Regularize Zone*’ and ‘*Real Space Refine Zone*’. Due to overall poor quality of the REC density, we did not refine it and it remains as a rigid body fit for model completeness. In the REC domain of all structures and in the PI domain of structure S3, there are regions of density that remain unmodeled. Final model validation and statistics were obtained with Phenix.

## 2-Aminopurine assay to detect unstacked nucleotides

Target strand oligonucleotides including a 2-aminopurine base in position 1 (5′-GACGCATACAGATGAGACG**A**CAAAGCACTAC-3′ – bold font shows the modification insertion, underlined nucleotides are the PAM), position 2 (5′-GACGCATACAGATGAGAC**G**ACAAA − GCACTAC-3′), position 4 (5′-GACGCATACAGATGAG**A**CGACAAAGCACTAC-3′) and position 13 (5′-GACGCAT**A**CAGATGAGACGACAAAGCACTAC-3′) downstream of the PAM sequence in the protospacer region, were obtained from IDT. Reactions (in triplicate, 25°C) were assembled and measured every 75 s over 2 hours using a Biotek Cytation 5 imaging reader with excitation wavelength 320 nm and emission wavelength 370 nm. Reading at 75 sec point was used for analysis. Reading of triplicates were averaged together. All measurements were normalized to the signal from double stranded DNA alone and measurement error was propagated using the following formula: $\frac{{\underset{\raise0.15em\hbox{$\smash{\scriptscriptstyle-}$}}{x} }}{{\underset{\raise0.15em\hbox{$\smash{\scriptscriptstyle-}$}}{y} }}\sqrt {{{{( {\frac{{{{\sigma }_x}}}{{\underset{\raise0.15em\hbox{$\smash{\scriptscriptstyle-}$}}{x} }}} )}}^2} + {{{( {\frac{{{{\sigma }_y}}}{{\underset{\raise0.15em\hbox{$\smash{\scriptscriptstyle-}$}}{y} }}} )}}^2}}$ where $\underset{\raise0.3em\hbox{$\smash{\scriptscriptstyle-}$}}{x}$ is the average of Cas12a-RNA-DNA or Cas12a-DNA signal; $\underset{\raise0.3em\hbox{$\smash{\scriptscriptstyle-}$}}{y}$ is the average of DNA only signal; ${{\sigma }_x}$ is a standard deviation for Cas12a-RNA-DNA or Cas12a-DNA signal; ${{\sigma }_y}$ is a standard deviation for DNA only signal.

### DNA bend and twist calculations

We quantified the local DNA bending and unwinding simultaneously, using an established inter-helical Euler angle approach (22,23). This method measures the bending magnitude (β_h, 0° ≤ β_h ≤ 180°), bending direction (γ_h, –180° ≤ γ_h ≤ 180°), and helical twist (ζ_h, –180° ≤ ζ_h ≤ 180°) between two helices (H1 and H2) across a junction (J) containing one or multiple base pairs. We defined the PAM sequences as H1, the spacer sequences two base pairs away from PAM as H2 and the two base pairs immediately adjacent to PAM in the spacer as J. Two idealized B-form DNA helices, each comprising 3 base pairs, were constructed using the 3DNA software (24) and superimposed onto H1 and H2, respectively. This alignment enabled us to determine the relative orientation of H1 to H2, quantified by the parameters β_h, γ_h, and ζ_h. The underwinding angle of the helix was calculated by *N* × 36° – ζ_h, where *N* is the number of base pairs in the J. Using this method, bending angle for Cas9 interrogation complex with bent DNA and closed protein conformation had a bend angle ∼69° and unwinding of ∼66°, while the linear DNA conformation in an open protein conformation had a bend angle of ∼38° and unwinding of ∼16°.

### Figure preparation

The comparison of PI domains was conducted after structure alignment with SSM superpose in Coot using the protein chain. For figure visualization, models were aligned with Chimera X (1.7.1) (25) matchmaker with restriction to residues 719–1307 for both the reference and moving model. Maps were aligned together with models with the same procedure. DNA-protein contacts were listed with Chimera X ‘contacts’ command. Figures were prepared with Chimera X (1.7.1). Chromatogram of complex purification and 2-AP assay results were plotted using GraphPad Prism 8.

## Results

### Cryo-EM structures of a crosslink-stabilized Cas12a:guide RNA:non target DNA complex

To investigate target search by a Cas12a-guide RNA complex, we designed DNA substrates bearing a 5′-TTTG-3′ PAM sequence but lacking any base pairing complementarity with the guide RNA (Figure 1B). We captured transient Cas12a RNP association with this substrate by introducing a disulfide crosslink between mutated amino acid N551C in Cas12a and an N4-cystamine-cyt(7) DNA modification. Control experiments confirmed that N551C mutation in Cas12a did not disrupt its ability to cleave target DNA (Supplementary Figure S1A). Formation of the disulfide crosslink between Cas12a and the modified DNA was confirmed by denaturing, non-reducing SDS-PAGE analysis (Supplementary Figure S1B,C). Further control experiments confirmed that Cas12a N551C cleaves DNA under crosslinking conditions (Supplementary Figure S1D) at levels comparable to WT Cas12a (Supplementary Figure S1A,D) showing that crosslinking does not interfere with Cas12a activity.

Cryo-EM analysis of the cross-linked sample revealed three distinct molecular structures (Supplementary Figure S1E, Supplementary Figure S2, Table 1). Conformations of the protein in each model are similar to each other and resemble Cas12a-guide RNA binary complexes (26,27). In all structures, the PAM sits in the Cas12a PAM-binding pocket (Figure 1C). Clear density corresponding to the crosslinking disulfide bridge between the protein and the DNA is seen in all conformations (Figure 1D). Consistent with the flexibility of the recognition (REC) lobe in both Cas12a and Cas9 noted in prior studies (6–8,26,28–32) we observed poor density for the REC domain in the structures determined here. Due to inherent flexibility, the resolution of the PI domain is lower than that of the stable NUC lobe core (Supplementary Figure S3) (26,30–32).

**Table 1.** CryoEM data collection, refinement and validation statistics

Data collection			
sample	Structure 1 PDB 9CJH EMD-45 631	Structure 2 PDB 9CJI EMD-45 632	Structure 3 PDB 9CJJ EMD-45 633
Magnification	36 000	36 000	36 000
Voltage (kV)	200	200	200
Electron dose (e-/Å^2^)	50	50	50
Defocus range (μm)	−0.8 to 2	−0.8 to 2	−0.8 to 2
Pixel size (Å)	1.115	1.115	1.115
**3D reconstruction**			
Raw images	1364	1364	1364
Initial particles	1 929 565	1 929 565	1 929 565
Final particles	20 031	27 147	27 031
Map resolution (Å)	3.6	3.4	3.5
FSC threshold	0.143	0.143	0.143
**Model refinement**			
Initial model used	Structure 2	5B43	Structure 2
Model resolution	3.82	3.67	3.77
FSC threshold	0.5	0.5	0.5
Model composition Nonhydrogen atoms Protein residues Nucleotide Ligands	11 200 1236 57 1	11 477 1244 67 1	11 240 1240 55 1
B factors (mean, Å^2^) Protein Nucleotide Ligand	63.91 94.49 97.95	62.5 101.99 76.23	58.76 71.14 52.20
R.m.s deviations Bond length (Å) Bond angles (°)	0.003 0.563	0.003 0.546	0.003 0.531
**Validation**			
MolProbity score	1.73	1.60	1.76
Clash score	9.99	9.38	13.53
Rotamer outlier (%)	0.83	0.83	1.11
Ramachandran statistics (%)- Favored Allowed Outlier	96.74 3.26 0	97.5 2.5 0	97.56 2.44 0
Rama-Z score, whole (r.m.s Rama-Z)	−0.88 0.23	−0.08 0.24	−0.6 0.24
Map CC (box)	0.67	0.69	0.69
Map CC (mask)	0.65	0.66	0.66

### Movement of the PAM interaction domain induces DNA bending

**Figure 2.**
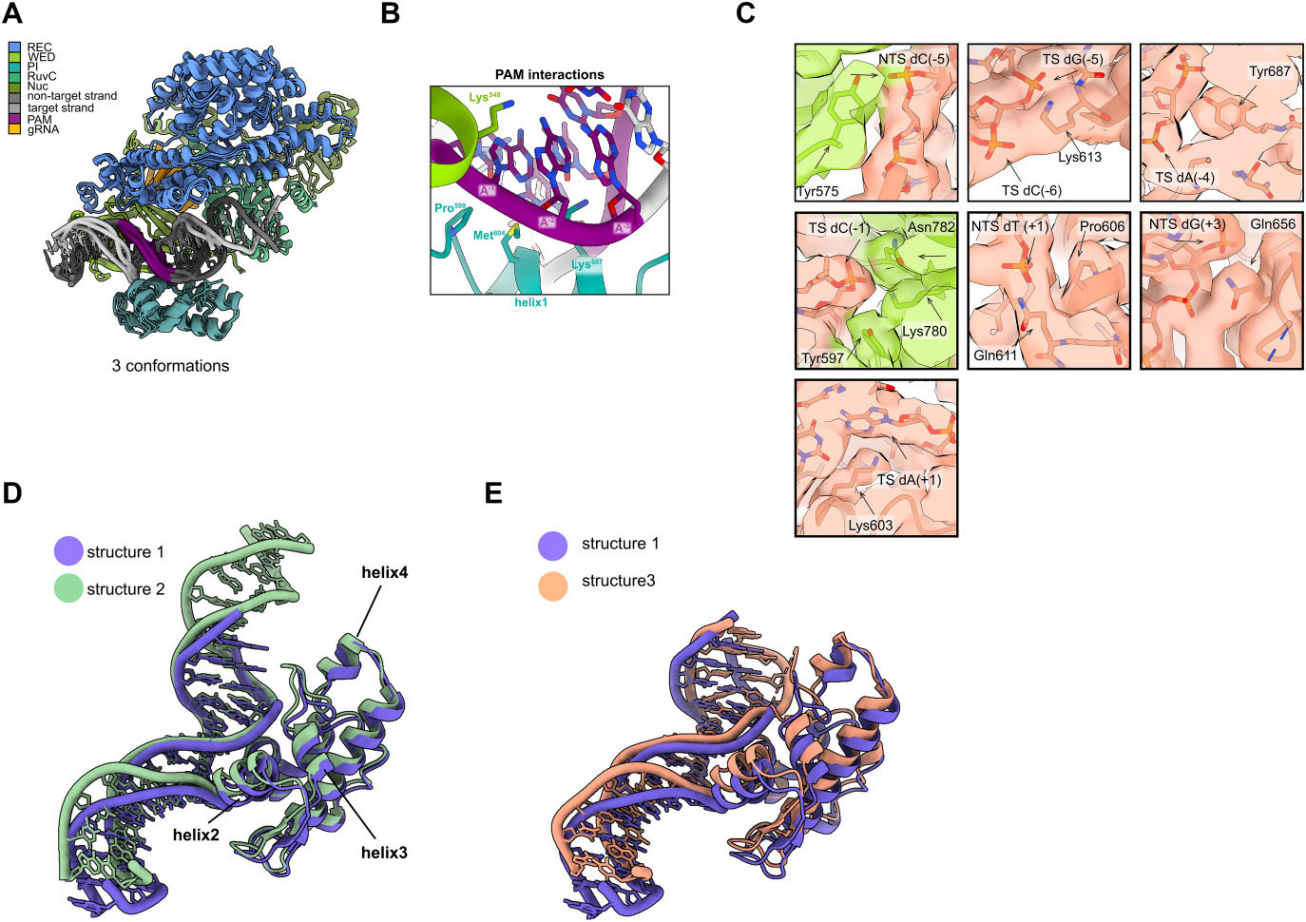
Cas12a–DNA interactions and PI domain movements (**A**) Global protein alignment of all structures. (**B**) Detail of PAM recognition interactions. View from the target strand side. (**C**) Details of Cas12a–DNA interactions in structure 3. TS stands for target strand and NTS for non-target strand. Map shown at level 0.0125. (**D**, **E**) Close-up of the PI domain and DNA conformational rearrangements.

We analyzed Cas12a–DNA interactions in our cryoEM structures to assess their similarity to prior Cas12a complexes containing target-engaged Cas12a RNPs (2,7,30,33). In all structures, the DNA sits in a positively charged groove of PI and WED domains. In structure 2, Pro599, Met604 and Lys607 of the PAM-interacting (PI) domain and Lys548 of the Wedge (WED-II) domain form contacts with the PAM directly adjacent to the crosslink position (Figure 2A,B) (2,3). PAM recognition is a prerequisite to target interrogation. In structure 1 and 3, key interactions between Lys548 and the PAM are absent. Adenosine in position (–2) on the target strand is 4.5–4.6 Å away from Lys548 side chain, placing it out of range for hydrogen bonding. In structures 2 and 3, there are additional protein–DNA interactions that help to stabilize the DNA position in the complex. The region upstream to the PAM motif is stabilized by interactions between the WED domain residue Tyr575 with the backbone of the non-target strand, as well as by interaction between PI domain residue Lys613 with the backbone of the target strand (Figures 2C, S4). In structure 1, there is no density for the loop 571–576 containing the Tyr575, and no side chain density for Lys613 precluding further interpretation of those regions (Supplementary Figure S4A). In addition, in all structures, Tyr687 forms a hydrogen bond with the backbone of the target strand in the PAM region, further stabilizing PAM - PI domain interactions (Figures 2C, S4A). The phosphate of the last PAM nucleotide (–1) on the target strand in all structures is contacted by the Tyr597, Lys780 and Asn782 residues through polar interactions (Figures 2C, S4A). Previously this group of residues was reported to facilitate the rotation of the (–1) phosphate that contributes to flipping of the (+1) base (26,30). Furthermore, Lys603 forms hydrogen bonding with the base of (+1) nucleotide on the target strand (Figure 2C). It has been reported previously that this residue contributes to the disruption of the (+1) base pairing, favoring base flipping (30). In our structure, base pairing of the (+1) base is not disrupted, potentially due to the stabilizing effect of Lys603. Downstream of the PAM region, the (+1) phosphate on the non-target strand is coordinated by Pro606 and Gln611. Gln656 interacts with the (+2) phosphate in structure 1, and with (+3) phosphate in structure 3 due to general PI domain shift towards the DNA and DNA bending, which brings (+3) phosphate closer to Gln656 (Figures 2C, S4A). The density for the Gln656 side chain is not observed for structure 2, preventing us from interpreting this region. These interactions as well as interactions with (–1) phosphate on the target strand may serve as stable connection points that determine the inflection point downstream of which DNA bends freely, resulting in three different DNA bending and underwinding conformations observed in this study. This flexibility may encourage separation of the NTS from the TS, culminating in base flipping and allowing interrogation of the target DNA strand. The observed bending may contribute to flipping of the second base (+2) on the target strand which may form a non-specific hydrogen bond with the second gRNA base in the seed region. Additionally, we observe a weaker density for the base (+2) of the non-target strand suggesting potential destabilization of this region.

**Figure 3.**
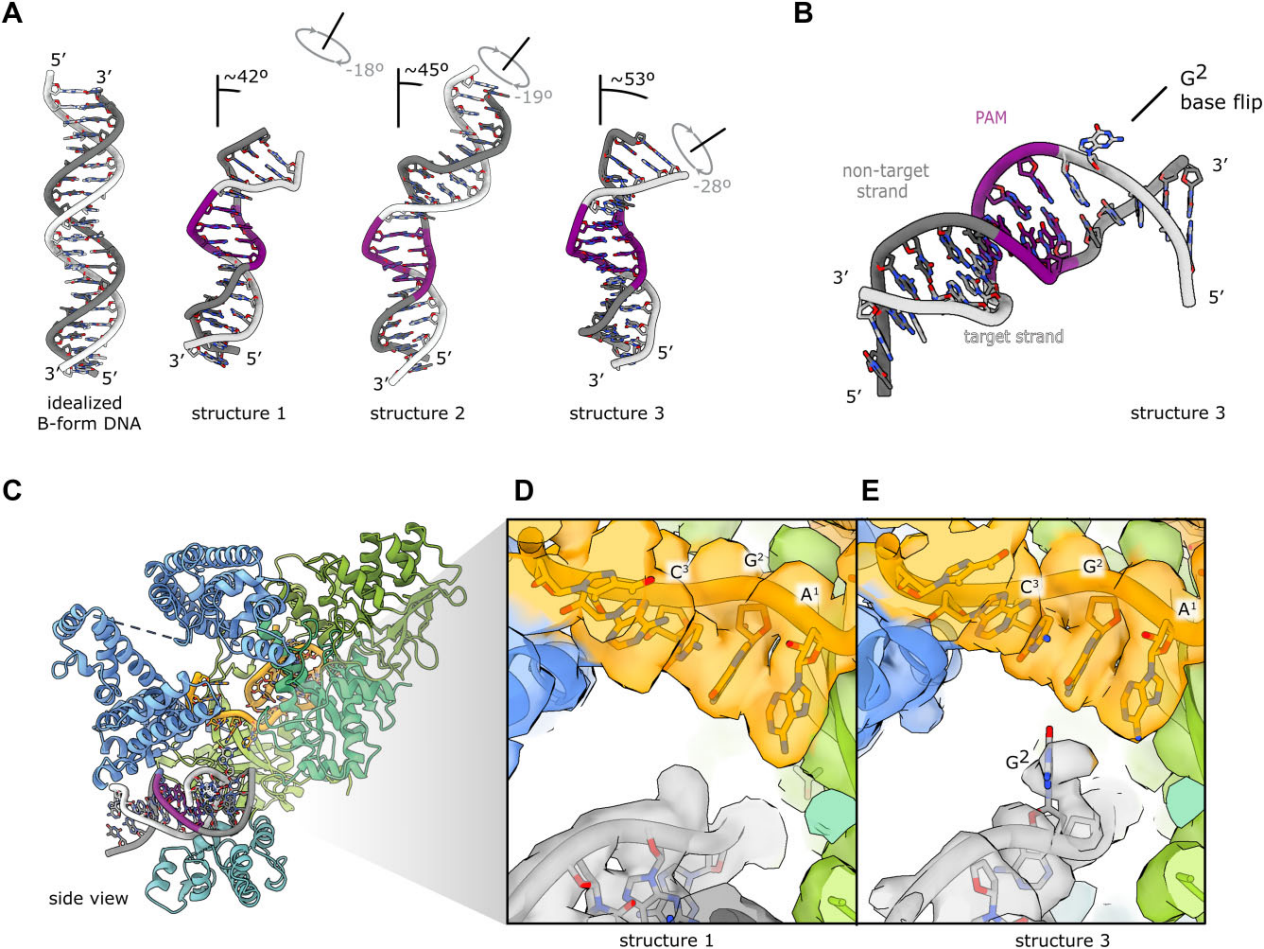
DNA bending and base flipping. (**A**) Comparison of B-form DNA with all DNA bent and underwound conformations. (**B**) View of structure 3 DNA showing flipped base. (**C**) Side view of structure 3. (**D**) Close-up of the density in proximity to the RNA seed sequence in structure 1. (**E**) Close-up of the density in proximity to the RNA seed sequence in structure 3. Maps are shown at 0.0125 level.

The C-alpha alignment of all three molecular models on a stable protein core (residues 719–1307) shows the PI domain of structure 2 minimally shifts towards the DNA. The shift is best visible for helix 2 (Supplementary Figure S4B). This shift is accompanied by an intermediate DNA conformation with a more pronounced bending and underwinding (45° bending and 19° underwinding versus 42° bending and 18° underwinding) than in structure 1 (Figures 2D, 3A). Structure 3 displays larger relative movement of the PI domain. Relative to structure 1 the entire PI domain of the structure 3-fold towards the DNA (Figure 2E), which is best observed for the C-terminal part of helix 1, helix 2 and less so for helix 5 (Supplementary Figure S4B). The result is a large bending and underwinding of the DNA of 53° and 28°, respectively (Figure 2E, 3A). The quality of density for helix 3 and 4 is low, preventing us from interpreting the movement of those regions. These results suggest that movements of the PI domain correlate with DNA bending and underwinding. We propose that structures 1 through 3 represent progression through a conformational landscape of protein movement and concomitant DNA bending.

### Cas12a bends DNA to induce helical distortion and base flipping for DNA interrogation

We quantified the relative distortion of DNA in crosslinked structures (Figure 3A). DNA in the candidate protospacer region is progressively bent and underwound from structures 1 to 3. In structure 3, which displays a DNA bend of 53° and is underwound by 28°, we observe a weak density outside of the double stranded region suggesting base unstacking and flipping (Figure 3B–E). The nucleobase in position 2 downstream of the PAM within the candidate protospacer region can be observed in two conformations, fully stacked and rotated out of its normal DNA base-pairing position (Figure 3A, E; Supplementary Figure S3). The corresponding nucleobase on the opposite strand (NTS) has a weaker density, suggestive of flexibility. The presence of full base-pairing without alternative base conformations in structures 1 and 2, where DNA distortion is less pronounced, suggests base flipping arises from protein-induced DNA bending.

**Figure 4.**
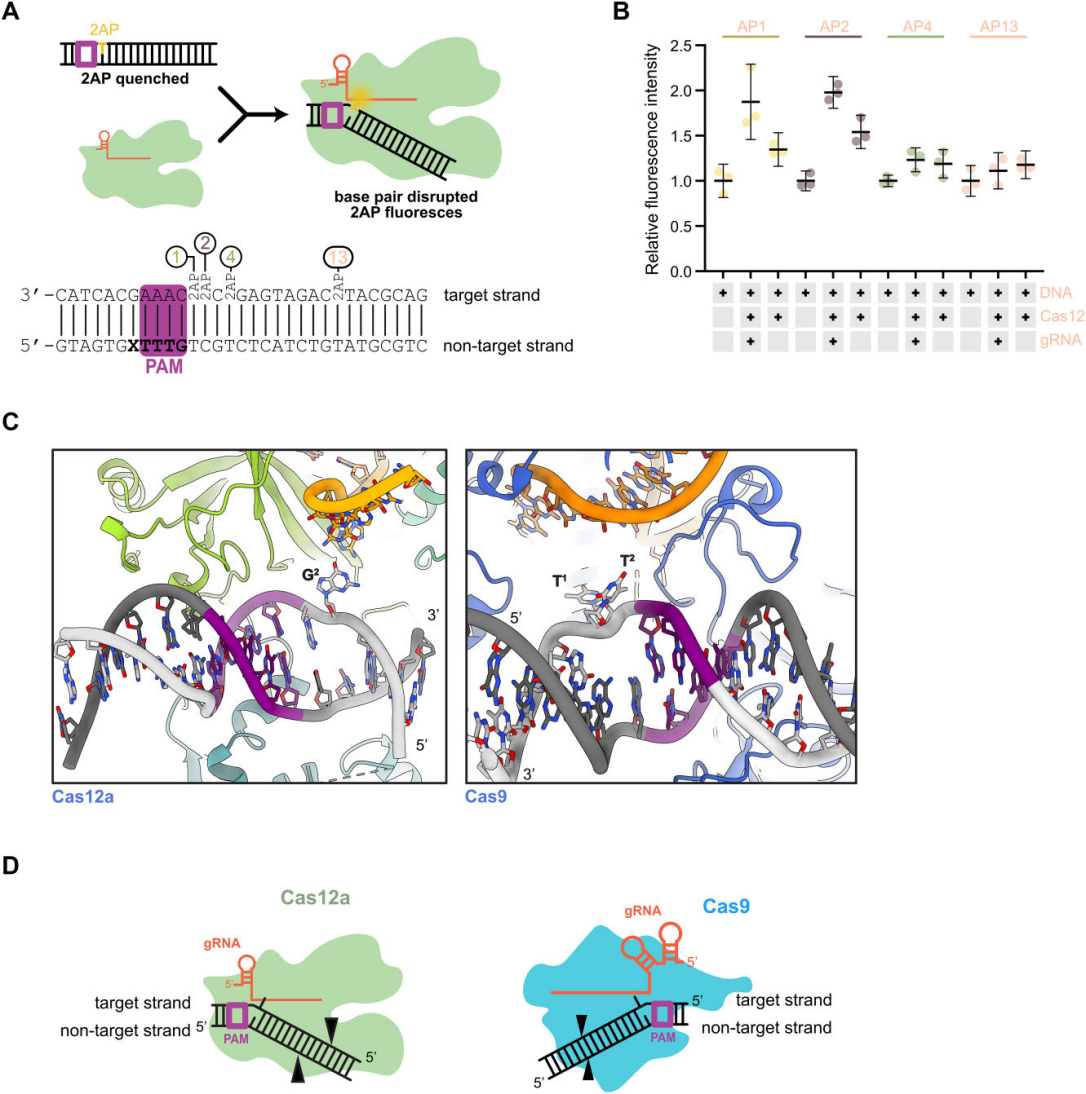
Cas12a interrogation mechanism. (**A**) Schematic representation of the 2-AP assay experimental design and expected outcomes. DNA sequence tested with positions of 2-AP insertion in all probes. (**B**) Graph showing triplicate fluorescence measurement for double stranded DNA, crosslinked Cas12–DNA–RNA complex and crosslinked Cas12-DNA control. The average of the triplicate signal normalized to the average of the double stranded DNA signal are plotted with error bars as described in methods. (**C**) Close-up of the PAM proximal region of Cas12a structure 3 (this study, left) and Cas9 interrogation complex (PDB: 7S36, right). (**D**) Schematic representation of Cas12a (left) and Cas9 (right) in DNA bending and base flipping state.

To investigate whether DNA base flipping occurs during Cas12a DNA interrogation, we employed a fluorescence-based biochemical assay. We designed DNA substrates containing single 2-aminopurine (2-AP) nucleotides at different positions relative to the PAM (Figure 4A). 2-Aminopurine is an analog of adenosine and guanosine and forms a Watson-Crick base pair with thymidine and a wobble configuration base pair with cytosine (34). This assay monitors the increase in fluorescence that occurs when 2-AP moves from a stacked, base-paired environment to an unstacked, single-stranded environment (Figure 4A). In control reactions with Cas12a crosslinked to DNA in the absence of guide RNA, we observed a small increase in fluorescence relative to the 2-AP-containing double stranded DNA alone, probably due to inherent flexibility of the protein in the apo state (35). We next conducted fluorescence detection assays using functional Cas12a RNPs crosslinked to a non-targeting sequence. We observed a pronounced increase in fluorescence for substrates with 2-AP at positions 1 or 2 downstream of the PAM. Only a small increase in fluorescence was observed for substrates with 2-AP at positions 4 and 13 (Figure 4B), which are far from the PAM and thus undisturbed during early DNA sequence probing. These data are consistent with our structural data suggesting Cas12a flips DNA bases independent of spacer complementarity during target interrogation. A recent study of the Cas9 DNA search mechanism provided evidence for solvent exposure of bases 1 and 2 adjacent to the PAM (8). Similarity between Cas9 and Cas12a suggests that base flipping induced by DNA bending is a common interrogation strategy for Class 2 CRISPR-Cas effectors.

## Discussion

In this study, we examined early stages of DNA interrogation by Cas12a. We trapped a normally transient DNA binding interaction to unravel the mechanism of initial DNA recognition before R-loop formation. Cryo-EM analysis of a cross-linked Cas12a-guide RNA-DNA complex revealed three distinct structural views representing a landscape of initial interactions between Cas12a and DNA. The DNA in each structure binds similarly to the PAM interacting (PI) domain, but differs in the degree of distortion from the canonical B-form helix. DNA bending appears to be induced by subtle conformational changes in the Cas12a PI domain. We observed the largest PI domain repositioning in structure 3, where helices and a bridging loop shift towards DNA. Protein motions accompany DNA bending and underwinding which culminates in a transient base flipping of the second nucleobase of the candidate protospacer region. The flipped base may serve to potentially initiate RNA-guided sequence interrogation. Utilizing crosslinking for structural analysis may inadvertently alter protein behavior. Therefore, we performed Cas12 cleavage assay to confirm the activity of our assembly. Furthermore, we confirmed conformational changes in a 2-AP assay, where we observed an increase in fluorescence for PAM-proximal, but not PAM-distal, bases. Analogous structures obtained for the Cas9 interrogation complexes showed that DNA bending correlates with differences in protein conformation (8). In the most distorted DNA structure, DNA was bent and underwound to 68° and 66°, respectively, exposing two bases adjacent to the PAM. Although Cas9 introduces more dramatic distortions than Cas12a, both are capable of flipping PAM-adjacent bases through a DNA bending mechanism to interrogate complementarity with the guide RNA. Our results demonstrate that Cas12, like Cas9 (8), induces conformational rearrangements to force DNA into a bent conformation. Ribonucleoprotein-mediated DNA bending causes base flipping and facilitates interrogation of the sequence after PAM binding. Whereas Cas9 interrogates the target sequence through REC and HNH domain movements (6,8,36), Cas12a repositions the PI and REC domains. DNA bending induced by Cas12 is less pronounced than Cas9-mediated bending. While there is density for a flipped base in two conformations in our structures, analogous Cas9 structures show unambiguous disruption of helical stacking(8). This suggests Cas9 more readily flips bases and as a consequence may reside longer at non-target sites. Conversely, Cas12 induces transient flipping in the absence of complementarity with the guide, which may enable faster discrimination between target and non-target sequences.

Cas9 searches for targets using both three-dimensional diffusion (37) and locally facilitated one-dimensional diffusion (38). In contrast, Cas12a searches for targets mainly through one-dimensional diffusion along the DNA and sometimes can even bypass a target site during DNA target searching (39). The cryo-EM model with less bent DNA in this study provides a potential physical explanation for the distinct search mode used by Cas12a. The frequency of Cas12a-induced base flipping may explain how often Cas12a bypasses target sites during 1D diffusion on the DNA.

Together, these data support a model in which Cas12a locates target sites within a genome by engaging PAM sequences and utilizing subtle PI domain conformational changes, along with REC domain movements, to bend and unwind DNA. Introduction of DNA distortion encourages base flipping, making PAM-adjacent nucleobase(s) accessible to potential base pairing with the guide RNA. Without guide complementarity, Cas12a releases the DNA and locates another PAM sequence.

Similarities between Cas9 and Cas12a DNA interrogation mechanisms imply convergent evolution of ATP-independent, RNA-mediated DNA interrogation. The evolution of Cas9 and Cas12a from different ancestral proteins (9) implies that transient DNA distortion induced by DNA bending may be a mechanism shared by RNA-guided enzyme families. Local DNA unwinding causes bases adjacent to the PAM to become unstacked and exposed in the direction of the guide RNA. This occurs despite opposite directionalities of the PAM with respect to the candidate protospacer region (Figure 4C). Both enzyme families use the biophysics of helix destabilization to enable RNA-guided target recognition (Figure 4D). In both their native (40,41) and biotechnological contexts (42,43), Cas9 and Cas12 survey numerous DNA sequences for the guide RNA complementarity, limiting efficiency by residency at off-target sites. We propose transient distortion of the PAM proximal region through DNA bending is an efficient biophysical solution to this problem. Cas effectors sample DNA locally before complete R-loop formation, enabling rapid dissociation from non-cognate spacers. These findings help explain why Class II CRISPR Cas effectors function even in eukaryotic genomes many times larger than those of their original prokaryotic hosts. Cas9 and Cas12 enzymes may differ in conformational sampling to tune speed and fidelity in their native contexts (40). Here, both structural and biochemical experiments were performed *in vitro*, and site-specific crosslinking was used to capture normally transient states. Although we confirmed activity under these conditions, future studies could analyze the effect of base flipping *in vivo* and leverage insights here to design improved genome editors. In addition, single molecule studies would further elucidate the dynamics of Cas12a DNA interrogation. These parameters may be utilized to engineer more efficient systems for genome editing efforts.

## Supplementary Material

gkae1192_Supplemental_Files

## Data Availability

Plasmid sequence map is available as a supplement. All structures are deposited in the PDB and EMDB with accession codes PDB ID: 9CJH, 9CJI, 9CJJ, and EMD-45631, EMD-45632, EMD-45633.

## Notes

Present address: Joshua C. Cofsky, Department of Biological Chemistry and Molecular Pharmacology, Harvard Medical School, Boston, MA 02115, USA.
