# Correction to ‘CRISPR-Cas12a bends DNA to destabilize base pairs during target interrogation’

**DOI:** 10.1093/nar/gkaf1150

**Published:** 2025-10-29

This is a correction to: Katarzyna M Soczek, Joshua C Cofsky, Owen T Tuck, Honglue Shi, Jennifer A Doudna, CRISPR-Cas12a bends DNA to destabilize base pairs during target interrogation, *Nucleic Acids Research*, Volume 53, Issue 2, 27 January 2025, https://doi.org/10.1093/nar/gkae1192

Grant number U19AI171110 has been removed from the **Funding** statement as this article is not within the scientific scope of the AViDD grant (discovery of antiviral drug compounds).

This change does not affect the results, discussion and conclusions presented in the article. The published article has been updated.

