# Supplementary material for: CRISPR-Cas12a bends DNA to destabilize base pairs during target interrogation
Source: Nucleic Acids Res. 2024 Dec 19;53(2):gkae1192. doi: 10.1093/nar/gkae1192 (PMC11754666; doi:10.1093/nar/gkae1192)
Supplement: gkae1192_Supplemental_Files [file gkae1192_supplemental_files.zip › Supplementary_Data_Soczek_etal_revision_seq.pdf]

Cas12a bends DNA to destabilize base pairs during target interrogation

Katarzyna M. Soczek[1,2,3], Joshua C. Cofsky[1,2#], Owen T. Tuck[2,4], Honglue Shi[1,5], Jennifer A. Doudna[1-7‡]

[1]Department of Molecular and Cell Biology, University of California, Berkeley; Berkeley, CA, USA; [2]Innovative Genomics Institute; University of California, Berkeley, CA, USA; [3]California Institute for Quantitative Biosciences (QB3), University of California, Berkeley, CA, USA; [4]Department of Chemistry, University of California, Berkeley; Berkeley, CA, USA; [5]Howard Hughes Medical Institute, University of California, Berkeley; Berkeley CA, USA; [6]Gladstone-UCSF Institute of Genomic Immunology; San Francisco, CA, USA; [7]Molecular Biophysics and Integrated Bioimaging Division, Lawrence Berkeley National Laboratory; Berkeley, CA, USA;

[#]Present address: Department of Biological Chemistry and Molecular Pharmacology, Harvard Medical School, Boston, Massachusetts 02115, USA

[‡]Corresponding author. Email: doudna@berkeley.edu

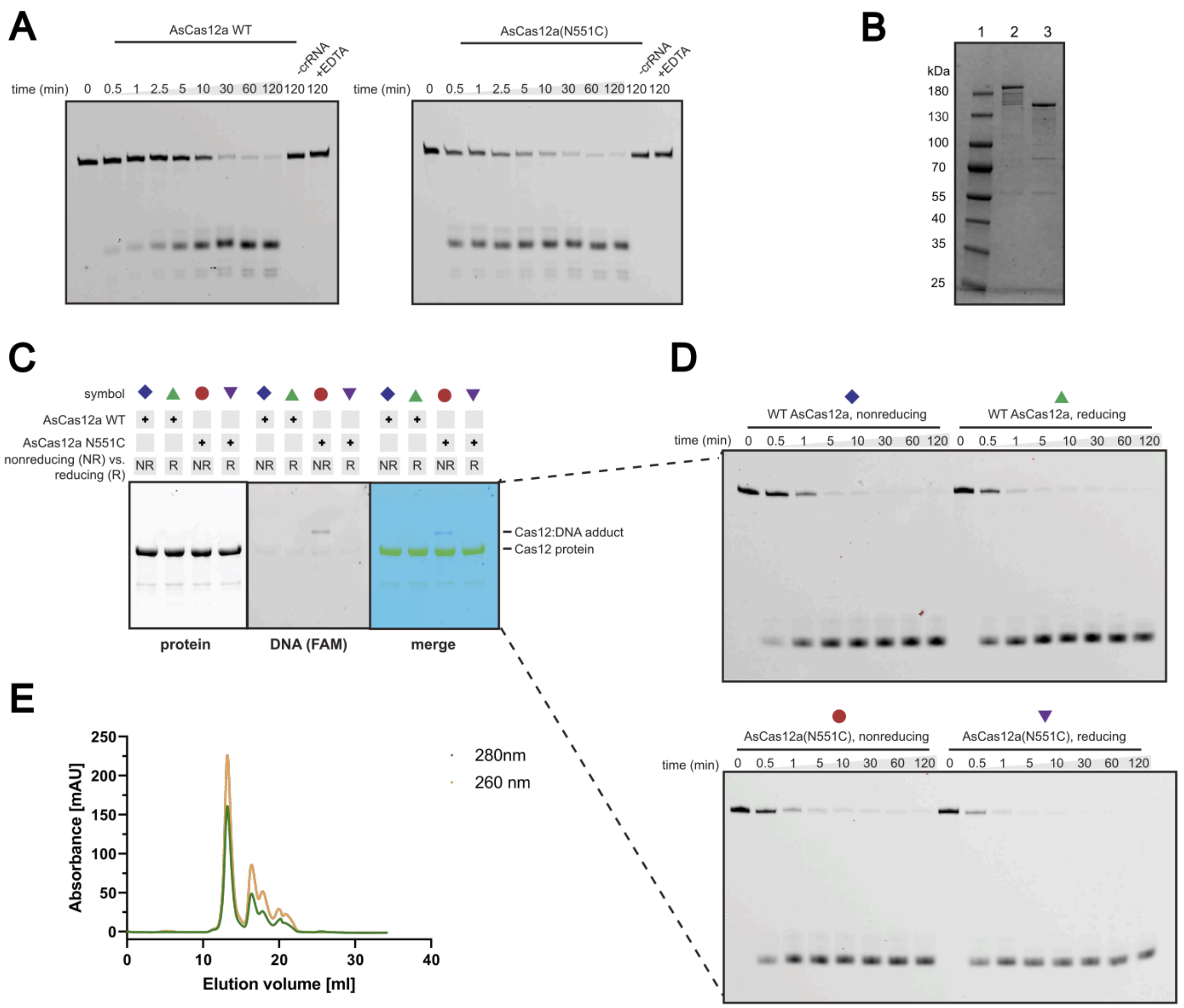

Supplementary Figure S1. Preparation of the crosslinked complex.

A. Cleavage assay with wild type and mutant AsCas12a with unmodified DNA (no crosslinking). **B.** Non-reducing SDS-PAGE gel showing efficiency of Cas12a(N551C)-DNA crosslinking in complex with RNA. Lane 1 protein ladder, lane 2 - crosslinked complex reaction, lane 3 - uncrosslinked protein alone. **C.** SDS-PAGE gel of the wild type and mutant AsCas12a with modified DNA under reducing and non-reducing conditions.**D**. Cleavage assay performed with samples from C. **E**. Size exclusion complex purification trace prior to cryoEM analysis.

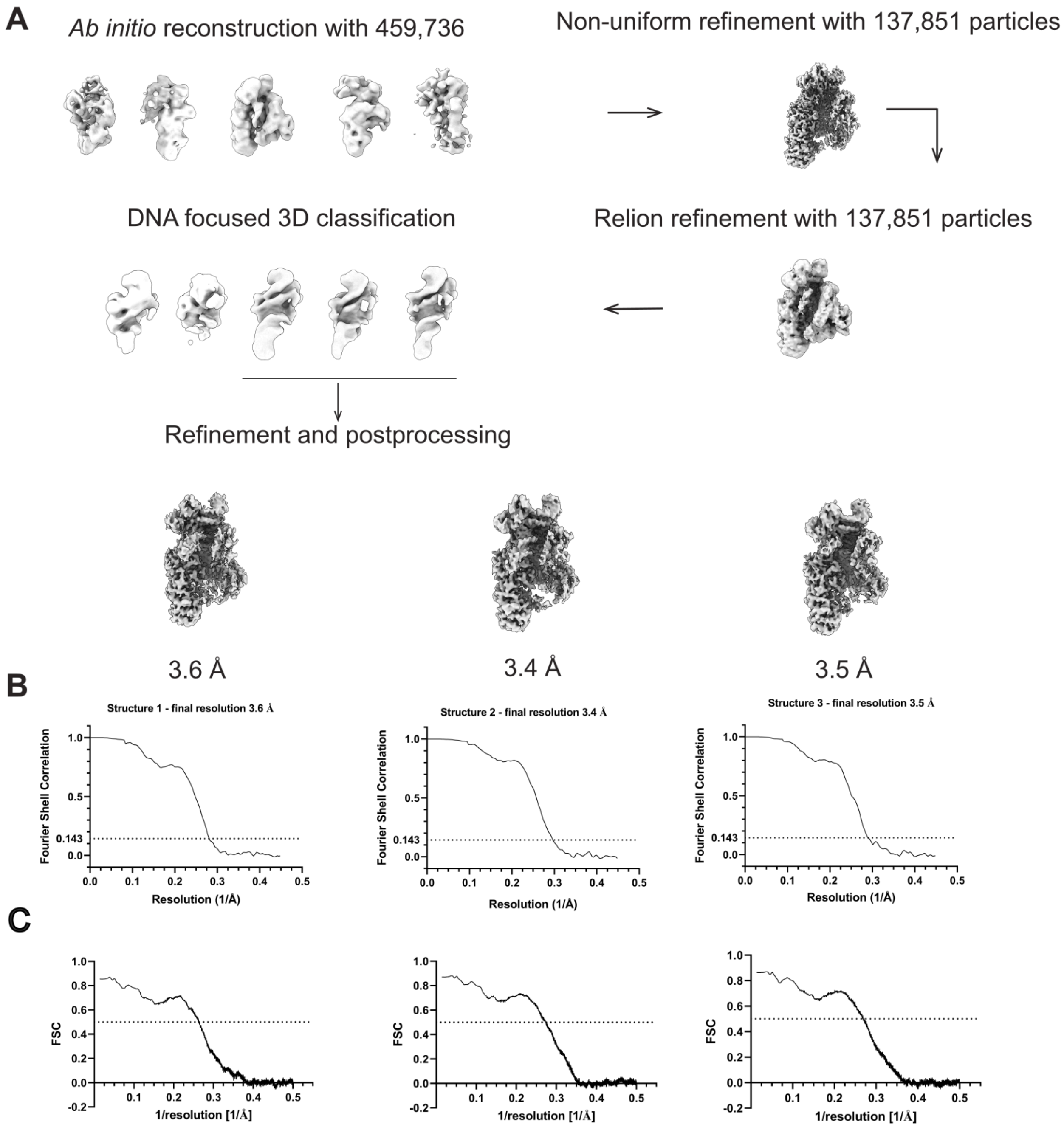

Supplementary Figure S2. CryoEM data processing.

A. Schematic of particle classification leading to final structures. **B.** FSC curves for each final map. **C.** Map-model FSC curves with masking, calculated in Phenix Mtriage with RELION postprocessed maps.

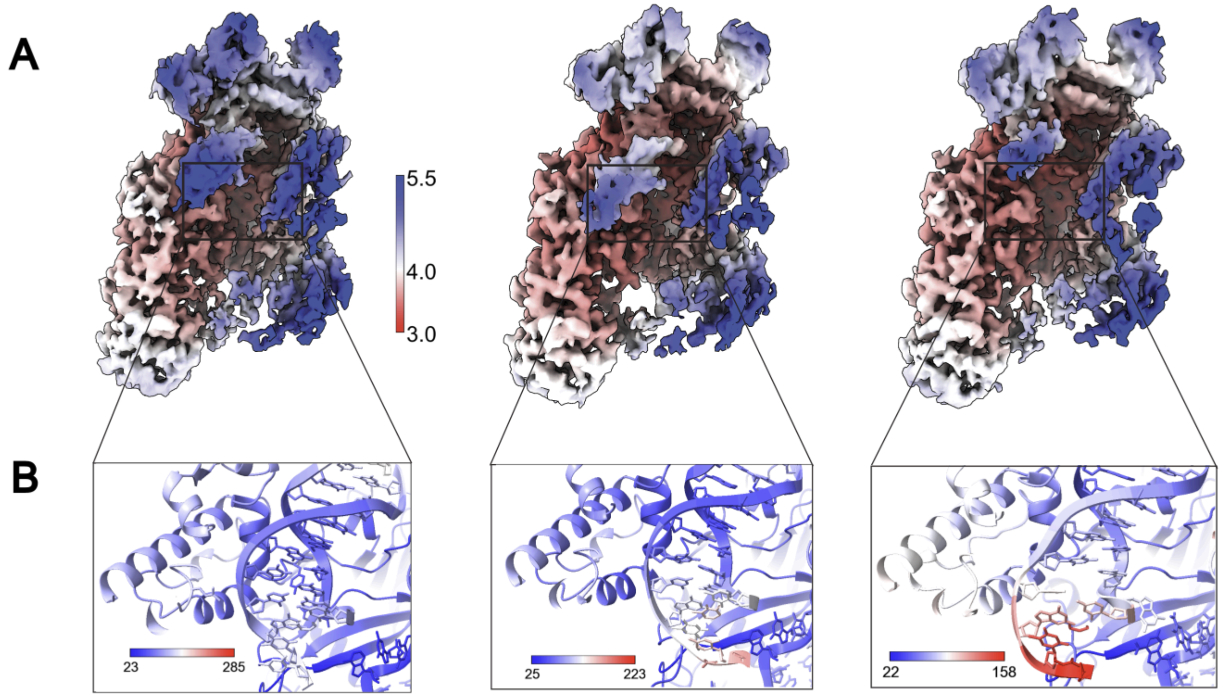

Supplementary Figure S3. Local structural variability.

A. Maps of all structures in this study colored based on local resolution calculated in RELION 5.

B. Close-up on PAM and a region of expected flipped base for all maps, colored based on B-factors.

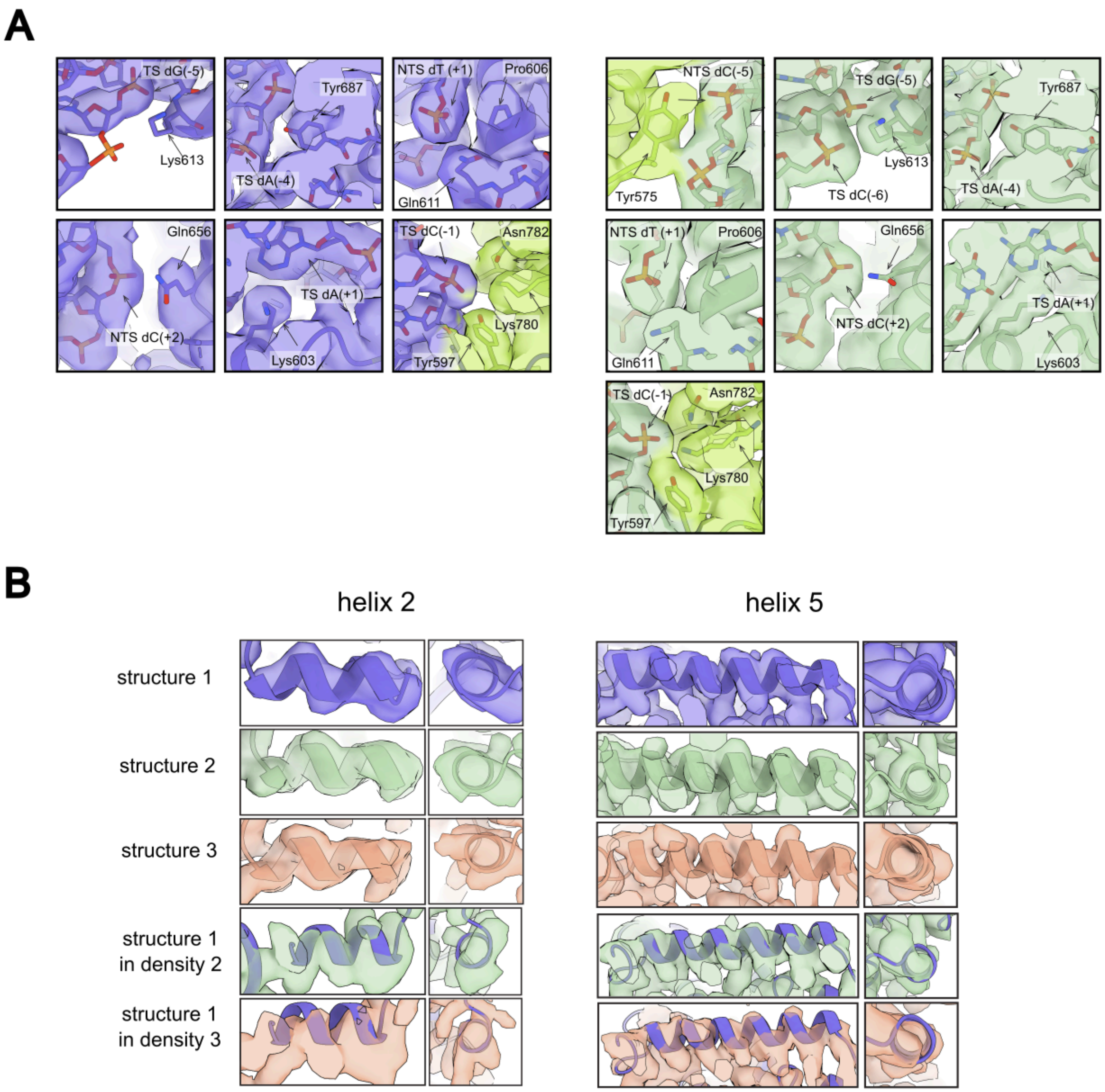

Supplementary Figure S4. Cas12a - DNA interactions and PI domain movement.

A. Protein-DNA interactions in structure 1 (left, purple). Protein-DNA interactions in structure 2 (right, green). Images taken at map level 0.0125. **B.** Images of helix 2 and 5 in their own densities for each structure, as well as images of structure 1 model in density of structure 2 and 3. Structure 1 model in density 2 and 3 for helix 2 are taken at a different angle than the images

of each model in its density. The angles were chosen to best show the fit to density and the movement. Images taken at map level 0.1.

Plasmid sequence for protein expression of AsCas12a N551C mutant (pJCC_99).

TTCTTGAAGACGAAAGGGCCTCGTGATACGCCTATTTTTATAGGTTAATGTCATGATAATAATGGTTTCTTAGACGTCAGGTGGCACTTTTCGGGGAAATGTGCGCGGAACCCCTATTTGTTTATTTTTCTAAATACATTCAAATATGTATCCGCTCATGAGACAATAACCCTGA
TAAATGCTTCAATAATATTGAAAAAGGAAGAGTATGAGTATTCAACATTTCCGTGTCGCCCTTATTCCCTTTTTTGCGGCATTTTGCCTTCCTGTTTTTGCTCACCCAGAAACGCTGGTGAAAGTAAAAGATGCTGAAGATCAGTTGGGTGCACGAGTGGGTTACATCGAACT
GGATCTCAACAGCGGTAAGATCCTTGAGAGTTTTCGCCCCGAAGAACGTTTTCCAATGATGAGCACTTTTAAAGTTCTGCTATGTGGCGCGGTATTATCCCGTGTTGACGCCGGGCAAGAGCAACTCGGTCGCCGCATACACTATTCTCAGAATGACTTGGTTGAGTACTC
ACCAGTCACAGAAAAGCATCTTACGGATGGCATGACAGTAAGAGAATTATGCAGTGCTGCCATAACCATGAGTGATAACACTGCGGCCAACTTACTTCTGACAACGATCGGAGGACCGAAGGAGCTAACCGCTTTTTTGCACAACATGGGGGATCATGTAACTCGCCTTG
ATCGTTGGGAACCGGAGCTGAATGAAGCCATACCAAACGACGAGCGTGACACCACGATGCCTGCAGCAATGGCAACAACGTTGCGCAAACTATTAACTGGCGAACTACTTACTCTAGCTTCCCGGCAACAATTAATAGACTGGATGGAGGCGGATAAAGTTGCAGGACC
ACTTCTGCGCTCGGCCCTTCCGGCTGGCTGGTTTATTGCTGATAAATCTGGAGCCGGTGAGCGTGGGTCTCGCGGTATCATTGCAGCACTGGGGCCAGATGGTAAGCCCTCCCGTATCGTAGTTATCTACACGACGGGGAGTCAGGCAACTATGGATGAACGAAATAGA
CAGATCGCTGAGATAGGTGCCTCACTGATTAAGCATTGGTAACTGTCAGACCAAGTTTACTCATATATACTTTAGATTGATTTAAAACTTCATTTTTAATTTAAAAGGATCTAGGTGAAGATCCTTTTTGATAATCTCATGACCAAAATCCCTTAACGTGAGTTTTCGTTCCACTG
AGCGTCAGACCCCGTAGAAAAGATCAAAGGATCTTCTTGAGATCCTTTTTTTCTGCGCGTAATCTGCTGCTTGCAAACAAAAAAACCACCGCTACCAGCGGTGGTTTGTTTGCCGGATCAAGAGCTACCAACTCTTTTTCCGAAGGTAACTGGCTTCAGCAGAGCGCAGA
TACCAAATACTGTCCTTCTAGTGTAGCCGTAGTTAGGCCACCACTTCAAGAACTCTGTAGCACCGCCTACATACCTCGCTCTGCTAATCCTGTTACCAGTGGCTGCTGCCAGTGGCGATAAGTCGTGTCTTACCGGGTTGGACTCAAGACGATAGTTACCGGATAAGGCGC
AGCGGTCGGGCTGAACGGGGGGTTCGTGCACACAGCCCAGCTTGGAGCGAACGACCTACACCGAACTGAGATACCTACAGCGTGAGCTATGAGAAAGCGCCACGCTTCCCGAAGGGAGAAAGGCGGACAGGTATCCGGTAAGCGGCAGGGTCGGAACAGGAGAG
CGCACGAGGGAGCTTCCAGGGGGAAACGCCTGGTATCTTTATAGTCCTGTCGGGTTTCGCCACCTCTGACTTGAGCGTCGATTTTTGTGATGCTCGTCAGGGGGGCGGAGCCTATGGAAAAACGCCAGCAACGCGGCCTTTTTACGGTTCCTGGCCTTTTGCTGGCCT
TTTGCTCACATGTTCTTTCCTGCGTTATCCCCTGATTCTGTGGATAACCGTATTACCGCCTTTGAGTGAGCTGATACCGCTCGCCGCAGCCGAACGACCGAGCGCAGCGAGTCAGTGAGCGAGGAAGCGGAAGAGCGCCTGATGCGGTATTTTCTCCTTACGCATCTGT
GCGGTATTTCACACCGCAATGGTGCACTCTCAGTACAATCTGCTCTGATGCCGCATAGTTAAGCCAGTATACACTCCGCTATCGCTACGTGACTGGGTCATGGCTGCGCCCCGACACCCGCCAACACCCGCTGACGCGCCCTGACGGGCTTGTCTGCTCCCGGCATCC
GCTTACAGACAAGCTGTGACCGTCTCCGGGAGCTGCATGTGTCAGAGGTTTTCACCGTCATCACCGAAACGCGCGAGGCAGCTGCGGTAAAGCTCATCAGCGTGGTCGTGAAGCGATTCACAGATGTCTGCCTGTTCATCCGCGTCCAGCTCGTTGAGTTTCTCCAGA
AGCGTTAATGTCTGGCTTCTGATAAAGCGGGCCATGTTAAGGGCGGTTTTTTCCTGTTTGGTCACTGATGCCTCCGTGTAAGGGGGATTTCTGTTCATGGGGGTAATGATACCGATGAAACGAGAGAGGATGCTCACGATACGGGTTACTGATGATGAACATGCCCGGTTA
CTGGAACGTTGTGAGGGTAAACAACTGGCGGTATGGATGCGGCGGGACCAGAGAAAAATCACTCAGGGTCAATGCCAGCGCTTCGTTAATACAGATGTAGGTGTTCCACAGGGTAGCCAGCAGCATCCTGCGATGCAGATCCGGAACATAATGGTGCAGGGCGCTGAC
TTCCGCGTTTCCAGACTTTACGAAACACGGAAACCGAAGACCATTCATGTTGTTGCTCAGGTCGCAGACGTTTTGCAGCAGCAGTCGCTTCACGTTCGCTCGCGTATCGGTGATTCATTCTGCTAACCAGTAAGGCAACCCCGCCAGCCTAGCCGGGTCCTCAACGACA
GGAGCACGATCATGCGCACCCGTGGCCAGGACCCAACGCTGCCCGAGATGCGCCGCGTGCGGCTGCTGGAGATGGCGGACGCGATGGATATGTTCTGCCAAGGGTTGGTTTGCGCATTCACAGTTCTCCGCAAGAATTGATTGGCTCCAATTCTTGGAGTGGTGAAT
CCGTTAGCGAGGTGCCGCCGGCTTCCATTCAGGTCGAGGTGGCCCGGCTCCATGCACCGCGACGCAACGCGGGGAGGCAGACAAGGTATAGGGCGGCGCCTACAATCCATGCCAACCCGTTCCATGTGCTCGCCGAGGCGGCATAAATCGCCGTGACGATCAGCG
GTCCAATGATCGAAGTTAGGCTGGTAAGAGCCGCGAGCGATCCTTGAAGCTGTCCCTGATGGTCGTCATCTACCTGCCTGGACAGCATGGCCTGCAACGCGGGCATCCCGATGCCGCCGGAAGCGAGAAGAATCATAATGGGGAAGGCCATCCAGCCTCGCGTCGCG
AACGCCAGCAAGACGTAGCCCAGCGCGTCGGCCGCCATGCCGGCGATAATGGCCTGCTTCTCGCCGAAACGTTTGGTGGCGGGACCAGTGACGAAGGCTTGAGCGAGGGCGTGCAAGATTCCGAATACCGCAAGCGACAGGCCGATCATCGTCGCGCTCCAGCGA
AAGCGGTCCTCGCCGAAAATGACCCAGAGCGCTGCCGGCACCTGTCCTACGAGTTGCATGATAAAGAAGACAGTCATAAGTGCGGCGACGATAGTCATGCCCCGCGCCCACCGGAAGGAGCTGACTGGGTTGAAGGCTCTCAAGGGCATCGGTCGACGCTCTCCCT
TATGCGACTCCTGCATTAGGAAGCAGCCCAGTAGTAGGTTGAGGCCGTTGAGCACCGCCGCCGCAAGGAATGGTGCATGCAAGGAGATGGCGCCCAACAGTCCCCCGGCCACGGGGCCTGCCACCATACCCACGCCGAAACAAGCGCTCATGAGCCCGAAGTGGC
GAGCCCGATCTTCCCCATCGGTGATGTCGGCGATATAGGCGCCAGCAACCGCACCTGTGGCGCCGGTGATGCCGGCCACGATGCGTCCGGCGTAGAGGATCGAGATCTCGATCCCGCGAAATTAATACGACTCACTATAGGGAGACCACAACGGTTTCCCTCTAGTGC
CGGCTCCGGAGAGCTCTTTAATTAAGCGGCCGCCCTGCAGGACTCGAGTTCTAGAAATAATTTTGTTTAACTTTAAGAAGGAGATATACATATGAAATCTTCTCACCATCACCATCACCATCACCATCACCATGGTTCTTCTATGAAAATCGAAGAAGGTAAACTGGTAATCTG
GATTAACGGCGATAAAGGCTATAACGGTCTCGCTGAAGTCGGTAAGAAATTCGAGAAAGATACCGGAATTAAAGTCACCGTTGAGCATCCGGATAAACTGGAAGAGAAATTCCCACAGGTTGCGGCAACTGGCGATGGCCCTGACATTATCTTCTGGGCACACGACCGCT
TTGGTGGCTACGCTCAATCTGGCCTGTTGGCTGAAATCACCCCGGACAAAGCGTTCCAGGACAAGCTGTATCCGTTTACCTGGGATGCCGTACGTTACAACGGCAAGCTGATTGCTTACCCGATCGCTGTTGAAGCGTTATCGCTGATTTATAACAAAGATCTGCTGCCG
AACCCGCCAAAAACCTGGGAAGAGATCCCGGCGCTGGATAAAGAACTGAAAGCGAAAGGTAAGAGCGCGCTGATGTTCAACCTGCAAGAACCGTACTTCACCTGGCCGCTGATTGCTGCTGACGGGGGTTATGCGTTCAAGTATGAAAACGGCAAGTACGACATTAAA
GACGTGGGCGTGGATAACGCTGGCGCGAAAGCGGGTCTGACCTTCCTGGTTGACCTGATTAAAAACAAACACATGAATGCAGACACCGATTACTCCATCGCAGAAGCTGCCTTTAATAAAGGCGAAACAGCGATGACCATCAACGGCCCGTGGGCATGGTCCAACATC
GACACCAGCAAAGTGAATTATGGTGTAACGGTACTGCCGACCTTCAAGGGTCAACCATCCAAACCGTTCGTTGGCGTGCTGAGCGCAGGTATTAACGCCGCCAGTCCGAACAAAGAGCTGGCAAAAGAGTTCCTCGAAAACTATCTGCTGACTGATGAAGGTCTGGAA
GCGGTTAATAAAGACAAACCGCTGGGTGCCGTAGCGCTGAAGTCTTACGAGGAAGAGTTGGCGAAAGATCCACGTATTGCCGCCACTATGGAAAACGCCCAGAAAGGTGAAATCATGCCGAACATCCCGCAGATGTCCGCTTTCTGGTATGCCGTGCGTACTGCGGTG
ATCAACGCCGCCAGCGGTCGTCAGACTGTCGATGAAGCCCTGAAAGACGCGCAGACTAATTCGAGCTCGAACAACAACAACAATAACAATAACAACAACCTCGGGATCGAGGAAAACCTGTACTTCCAATCCAATGCAATGACCCAGTTCGAGGGGTTTACCAATCTGTA
TCAAGTGAGCAAGACGCTGCGCTTTGAACTGATCCCACAGGGAAAAACCTTAAAACATATTCAAGAGCAGGGCTTTATCGAAGAAGATAAGGCCCGTAATGACCATTACAAAGAGTTAAAGCCGATTATTGATCGTATCTACAAGACCTATGCGGACCAGTGCTTACAATTG
GTACAGCTTGATTGGGAGAACCTCTCTGCCGCCATCGATTCCTATCGTAAAGAAAAAACTGAAGAAACGCGCAACGCCCTGATTGAAGAGCAGGCCACCTATCGTAACGCGATTCATGACTATTTTATTGGCCGTACGGACAATCTGACGGACGCGATCAACAAGCGCCA
TGCGGAGATTTACAAAGGACTGTTTAAGGCTGAACTGTTCAATGGTAAGGTCCTTAAACAGCTTGGGACCGTCACAACGACGGAACATGAAAACGCGTTATTACGTAGCTTCGACAAGTTTACCACGTATTTCTCCGGCTTTTACGAAAATCGCAAAAACGTTTTCAGTGC
CGAGGATATTTCCACTGCTATCCCTCATCGCATTGTGCAAGACAACTTCCCAAAATTCAAAGAAAATTGTCATATCTTCACCCGCTTAATCACCGCTGTACCGTCCCTGCGTGAGCATTTCGAAAACGTGAAAAAGGCCATTGGTATCTTCGTGTCTACTTCGATTGAGGAG
GTATTTTCCTTTCCATTCTATAATCAGCTGCTGACCCAGACCCAAATTGATCTGTACAACCAGCTGCTTGGCGGTATTTCTCGTGAAGCAGGAACCGAAAAAATCAAAGGGTTGAACGAGGTGCTTAATCTGGCAATCCAGAAAAATGATGAAACCGCCCACATCATTGCTT
CGTTACCTCATCGTTTTATCCCGTTGTTCAAGCAAATTTTAAGTGATCGCAATACGCTGTCGTTTATTCTGGAAGAATTCAAAAGTGATGAAGAGGTAATTCAGTCGTTTTGCAAATATAAAACCCTGTTACGTAACGAAAATGTCCTGGAAACAGCCGAGGCTTTGTTTAACG
AACTGAATAGCATTGACCTGACGCATATCTTTATTAGCCACAAAAAATTAGAGACCATCTCATCAGCTCTGTGCGATCATTGGGATACACTGCGCAATGCGCTGTATGAACGTCGTATTTCGGAATTGACTGGCAAAATCACTAAAAGCGCGAAAGAGAAAGTACAGCGCTC
GCTTAAACATGAAGATATCAACCTGCAGGAGATCATCAGCGCCGCGGGTAAAGAACTGTCGGAGGCATTTAAACAGAAGACGAGCGAGATTCTGTCCCACGCACATGCCGCCTTAGACCAGCCGCTCCCGACCACTCTGAAGAAACAGGAAGAGAAAGAAATCCTTAAA
AGTCAACTGGACAGTTTACTGGGTCTCTATCATCTGCTGGATTGGTTTGCGGTAGACGAAAGCAATGAAGTGGATCCGGAGTTTAGTGCCCGTCTGACAGGAATCAAGCTGGAAATGGAGCCTTCGCTTAGCTTCTACAACAAAGCCCGCAATTATGCCACGAAAAAACC
CTATAGTGTCGAAAAATTTAAACTCAACTTTCAAATGCCGACCCTTGCGTCGGGCTGGGATGTCAACAAAGAAAAAtgtAACGGAGCTATTCTGTTCGTTAAAAATGGTCTGTACTACCTGGGCATCATGCCGAAACAGAAAGGTCGCTACAAAGCCCTTTCGTTCGAGCCC
ACGGAAAAAACAAGCGAAGGCTTCGACAAAATGTACTACGATTACTTTCCGGATGCAGCAAAAATGATCCCGAAATGTTCCACACAGCTGAAAGCCGTTACAGCACATTTTCAGACGCACACCACCCCCATCTTACTGTCCAACAATTTTATTGAACCGCTGGAGATTACTA
AAGAAATTTATGATTTGAACAATCCGGAAAAAGAGCCAAAAAAGTTTCAAACCGCCTACGCTAAAAAAACCGGGGATCAGAAAGGGTACCGCGAAGCGTTGTGCAAGTGGATTGATTTCACCCGCGATTTTCTCAGTAAATATACCAAGACTACCTCGATTGACCTGAGCT
CACTGCGCCCGAGCTCTCAATATAAGGATTTGGGTGAGTACTATGCTGAATTAAACCCTTTATTGTACCACATTTCTTTTCAGCGCATCGCCGAAAAGGAAATTATGGACGCAGTCGAAACCGGGAAACTGTACCTGTTCCAGATCTATAATAAGGACTTCGCCAAAGGACA
TCATGGCAAACCGAACCTGCACACCCTTTACTGGACCGGGCTTTTCTCTCCGGAAAATTTGGCGAAAACCTCGATCAAGCTTAACGGTCAAGCTGAGCTGTTTTACCGTCCAAAATCCCGCATGAAGCGCATGGCGCATCGTTTAGGTGAAAAAATGCTGAATAAGAAAC
TGAAAGATCAGAAAACCCCTATCCCGGATACCCTCTACCAGGAACTGTATGATTACGTGAACCATCGTCTCTCGCATGACCTGTCAGACGAAGCGCGTGCGTTACTGCCCAATGTAATCACAAAAGAAGTTTCGCATGAAATTATTAAAGATCGTCGTTTTACATCTGATAAA
TTCTTTTTTCATGTTCCGATCACCCTCAACTATCAGGCCGCAAACAGTCCAAGTAAGTTTAACCAGCGCGTTAATGCTTACCTGAAGGAACATCCGGAGACTCCGATTATTGGAATTGATCGCGGTGAACGTAATTTGATCTATATCACTGTGATCGATAGTACCGGTAAGATT
CTGGAGCAGCGCAGCTTGAACACAATTCAACAGTTTGATTATCAGAAAAAATTAGACAACCGCGAAAAAGAGCGCGTGGCTGCCCGTCAGGCGTGGTCTGTTGTCGGTACCATTAAAGATCTGAAGCAGGGCTATCTTTCTCAGGTTATTCACGAAATTGTAGATCTGATG
ATCCATTATCAGGCGGTTGTTGTGTTGGAGAATCTCAATTTCGGTTTTAAGAGTAAGCGCACAGGCATCGCTGAAAAAGCAGTTTATCAGCAGTTTGAAAAAATGCTGATCGACAAATTGAACTGTTTAGTTCTCAAAGATTACCCAGCGGAAAAGGTGGGCGGAGTGCTG
AATCCGTACCAATTAACGGATCAATTCACTTCCTTCGCAAAGATGGGTACCCAAAGCGGCTTTCTGTTCTATGTGCCGGCCCCGTATACCTCGAAAATCGATCCACTGACGGGCTTCGTAGATCCGTTCGTGTGGAAAACCATTAAAAATCATGAAAGTCGTAAACATTTTC
TCGAAGGCTTCGACTTCCTGCACTACGACGTGAAAACTGGCGATTTCATTCTGCATTTTAAAATGAACCGCAACCTTTCGTTTCAGCGCGGTCTGCCGGGCTTTATGCCGGCTTGGGACATTGTTTTTGAGAAAAATGAAACCCAGTTTGATGCTAAGGGCACTCCTTTCA
TCGCCGGTAAACGCATCGTACCTGTGATTGAAAACCATCGTTTTACAGGGCGTTACCGTGATTTATACCCGGCGAACGAATTGATCGCGCTGCTGGAGGAAAAGGGCATCGTTTTCCGTGACGGCTCCAATATTCTGCCGAAATTACTGGAAAACGACGATTCACACGCA
ATTGATACCATGGTCGCACTGATTCGCTCAGTCTTACAGATGCGTAACTCTAATGCAGCCACAGGAGAAGATTATATTAATTCGCCAGTCCGCGATTTGAACGGTGTTTGCTTCGACAGCCGTTTTCAGAATCCTGAATGGCCGATGGACGCTGATGCCAACGGAGCTTATC
ATATCGCCCTGAAAGGCCAGCTCCTGCTGAACCACCTGAAGGAAAGCAAAGATCTGAAATTGCAGAACGGCATTAGCAACCAGGACTGGTTAGCATACATCCAGGAACTGCGTAACTAATAACTAATTGGAAGTGGATAACGGATCCGCGATCGCGGCGCGCCACCTGGTG
GCCGGCCGGTACCACGCGTGCGCGCTGATCCGGCTGCTAACAAAGCCCGAAAGGAAGCTGAGTTGGCTGCTGCCACCGCTGAGCAATAACTAGCATAACCCCTTGGGGCCTCTAAACGGGTCTTGAGGGGTTTTTTGCTGAAAGGAGGAACTATATCCGGATATCCAC
AGGACGGGTGTGGTCGCCATGATCGCGTAGTCGATAGTGGCTCCAAGTAGCGAAGCGAGCAGGACTGGGCGGCGGCCAAAGCGGTCGGACAGTGCTCCGAGAACGGGTGCGCATAGAAATTGCATCAACGCATATAGCGCTAGCAGCACGCCATAGTGACTGGCGA
TGCTGTCGGAATGGACGATATCCCGCAAGAGGCCCGGCAGTACCGGCATAACCAAGCCTATGCCTACAGCATCCAGGGTGACGGTGCCGAGGATGACGATGAGCGCATTGTTAGATTTCATACACGGTGCCTGACTGCGTTAGCAATTTAACTGTGATAAACTACCGCAT
TAAAGCTTATCGATGATAAGCTGTCAAACATGAGAA
